# Playing the mirror game in virtual reality with an autonomous character

**DOI:** 10.1038/s41598-022-25197-z

**Published:** 2022-12-09

**Authors:** Joan Llobera, Valentin Jacquat, Carmela Calabrese, Caecilia Charbonnier

**Affiliations:** 1 Artanim Foundation, Meyrin, 1217 Switzerland; 2 Department of Electrical Engineering and Information Technology, University of Naples Federico II, 80125 Naples, Italy; 3 Faculty of Medicine, University of Geneva, Geneva, 1211 Switzerland

**Keywords:** Human behaviour, Engineering

## Abstract

Perceptual-motor synchronisation in human groups is crucial in many activities, from musical ensembles to sports teams. To this aim, the mirror game, where partners are asked to imitate each other’s movements or gestures, is one of the best available experimental paradigms to study how humans engage in joint tasks and how they tend to synchronise their behaviour. However, to date, virtual reality characters do not engage in motor synchronisation with human users. In this work, we explored to what extent an autonomous virtual character and a human that play the mirror game in virtual reality can synchronise their behaviour. We created a full-body version of the mirror game with an autonomous virtual character, whose movements were driven by a model based on coupled oscillators. Participants engaged in a joint imitation task with a virtual player animated with one of three options: a model that included a small coupling, a model with no coupling, or another human. Behavioural measures and subjective reports suggest that participants were unable to distinguish the condition of small coupling from the engagement with an avatar driven by another human participant.

## Introduction

Perceptual-motor synchronisation in human groups is crucial in many activities, from musical ensembles to sports teams^1^. People engaged in joint tasks tend to synchronise their behaviour^2^, often without being aware of it. This tendency has several behavioural and cognitive benefits. When humans synchronise their behaviour, they tend to adopt pro-social attitudes^3,4^, to improve the memory of the task^5^ and to improve the estimation of cooperative goals^6^. Under appropriate circumstances, experiments based on joint tasks are also useful to illuminate psychological factors such as commitment^7,8^. To this aim, the mirror game paradigm has become a reference task in the joint action literature. It represents a common exercise in movement therapy, where partners are asked to imitate each other’s movements or gestures and it is used to promote participants’ ability to enter and remain in a state of togetherness. It is one of the best available experimental paradigms to study how humans engage in joint tasks and how they tend to synchronise their behaviour. It has been used, to show that individuals have their own motor signatures^9,10^, and that individuals with similar motor signatures tend to coordinate better^9^. It is also one of the best available experimental paradigms used to study *sensorimotor communication*^11^.

A different stream of work is the modeling of behaviour synchronisation with methods derived from the dynamical systems literature. Networks of heterogeneous Kuramoto oscillators with nonlinear coupling^12,13^ represent a classic model used to describe the emergent rhythmic behaviour in an ensemble, such as typically found in people clapping in concert halls^14^. This modelling work has also shown that even in simple tasks like joint finger tapping, there are sophisticated interpersonal synchronisation mechanisms that emerge^15^ . These models have also been adapted to study the extent to which people performing joint tasks could adapt to virtual players. For example, Zhai et al.^16,17^ showed that a virtual player can synchronise its behaviour with a human in an imitation task. Lombardi et al.^18^ showed that this model could be extended to work on multiple agents, by using data captured with a specially designed computer mediated setup^19^. Lombardi et al.^18^ also showed that the behaviour of a human in such tasks can be learnt, even to the extent that they captured the individual motor signature of the particular human being modelled. The dynamics of such patterns have also been modelled for groups of more than two humans^20–23^.

A different use of the mirror game has been its adoption to study the physiological responses associated with *being in the zone*, and the enhanced feelings of togetherness associated with it^24^. This idea is associated with the feeling of flow^25^, where people report a loss of the sense of time, and a good connection with their environment and the activity in which they are involved. In this context it has been associated with the notion of optimal performance in sport and physical activities^26^.

Overall, despite the rich and growing literature on the topic, the study of motor coordination and joint action remains challenging due to its social nature. To understand how people coordinate in joint tasks, it is fundamental to design experiments where participants are asked to perform cooperative tasks. The need for two or more participants to engage in an experiment considerably limits the extent to which we can, for example, use brain imagery techniques to understand the neural mechanisms involved in social coordination. Another limitation of mirror game studies is that experiments do not involve full body interaction, but rather simplified representations such as, for example, a flat ping-pong game^27^, a simple slider^10,28^ or just two dots displayed on a screen^29^. As a consequence, it is still unclear to what extent the results and computational models that apply to uni-dimensional movements can generalise to full-body interaction.

**Figure 1:**
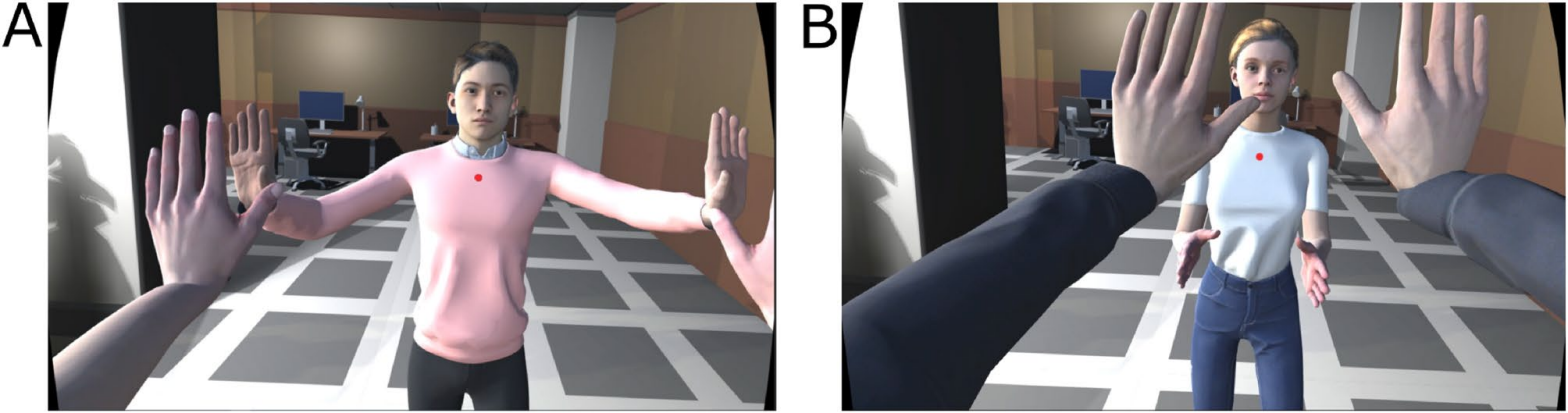
The virtual environment as seen from the perspective of two participants. In (**A**) we see an instant of time where behaviour is synchronous. In (**B**) we see the same virtual scene from the perspective of the other participant, when the behaviour has not synchronised. Note that the gender of the virtual character matched the gender reported at the beginning of the experiment by the other participant doing the task simultaneously.

In this work, we explored whether it is possible to replicate a full-body version of the mirror game paradigm^28,30^ with an autonomous virtual character. The extent to which autonomous VR characters engaged in joint motor tasks with humans can induce feelings of togetherness and flow has not been studied. For this purpose, we developed a full-body version of the mirror game^28,30^ for virtual reality (see Fig. 1, Supplementary Video 1) and asked participants to play the mirror game in a training trial, followed by three trials under different conditions. Results suggest an autonomous virtual character can elicit responses comparable to performing the task with another human, but only when there is coupling between the movements of the participant and the movements controlled by the computational model.

**Table 1:** Questionnaire responses. Wilcoxon tests comparing the graded responses to the *coupling* condition with the graded responses to the *no coupling* condition.

Question: During the last trial.	*No-coupling*	*Coupling*	Z score	p	*d*	Effect size
1. I did the task fluidly and smoothly	0.0	1.2	50.5	0.002	− 0.4	Medium
2. I had no problem to concentrate during the task	1.0	1.4	65.5	0.380	− 0.1	
3. I felt just the right amount of challenge	1.0	1.0	71.0	0.791	0.0	
4. I did not notice time passing	0.6	1.2	39.0	0.040	− 0.1	
5. I felt like the arms that moved when I moved were my own arms	0.4	1.8	25.0	0.002	− 0.3	Small
6. I felt as if the character in front of me was another person	1.4	0.4	108.5	0.050	0.3	Small
7. My movements influenced the movements of the character that was in front of me	− 0.1	1.3	101.5	0.012	− 0.4	Medium
8. The movements of the character in front of me influenced my own movements	1.8	0.9	49.5	0.037	0.3	Small
9. The character in front of me moved exactly like me, as if I was looking at a mirror	− 1.5	0.3	48.0	0.000	− 0.5	Medium
10. When the character in front of me moved, I felt the instinct to move	1.1	1.0	127.0	0.504	0.1	

The values reported are the median values for each condition, the Z score, the p value and Cliff’s delta. The effect size is estimated from this last metric.

**Figure 2:**
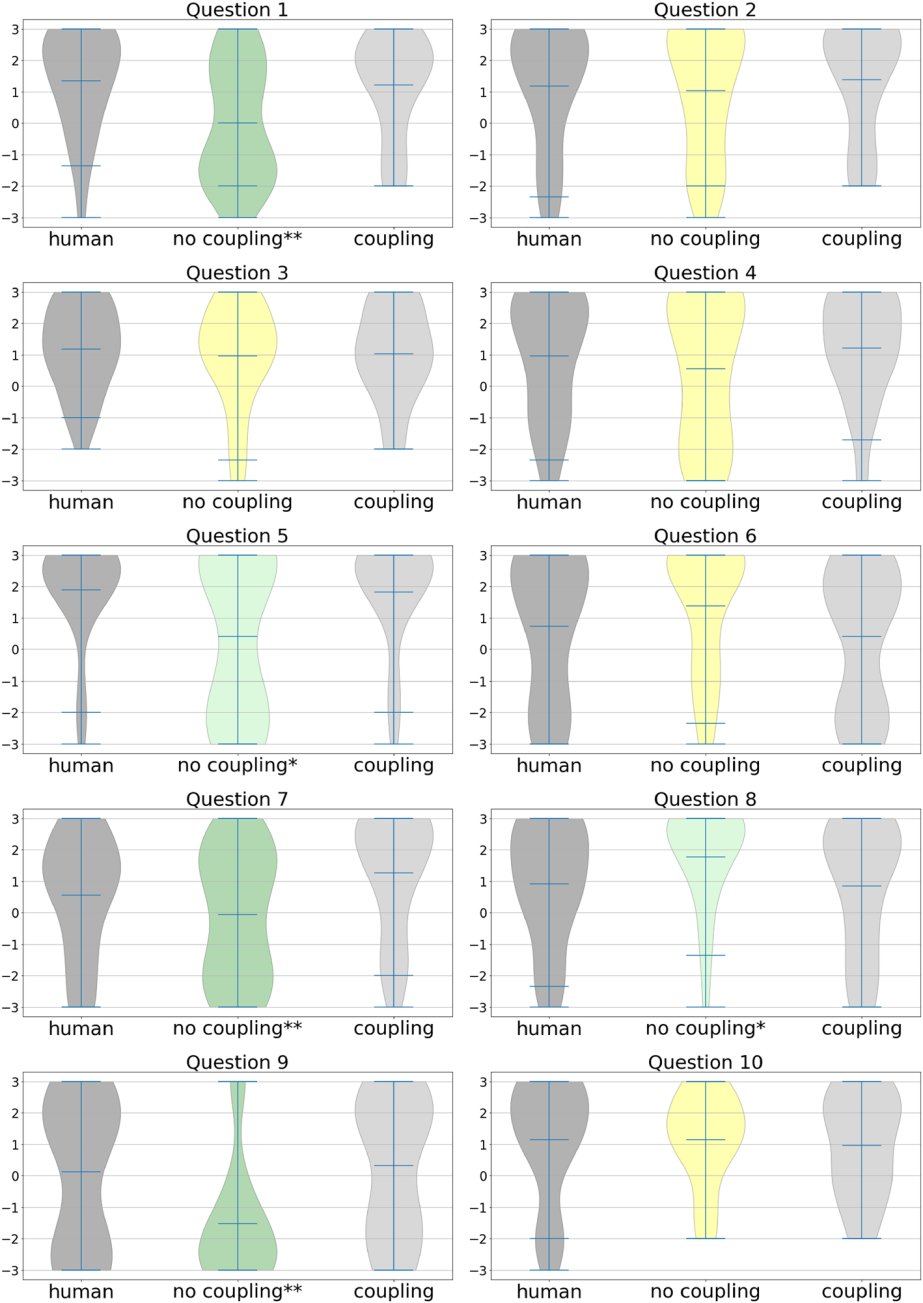
Violin plots of the graded responses to the questions. First column shows responses when participants did the task with another human (dark grey). No question showed a significant difference when comparing another human with the *coupling* condition. The second column shows responses when participants did the task with an automated virtual human in the *no coupling* condition. Responses in green are the responses which are significantly different from the *coupling* condition (p< 0.05). Light green and * in the label indicate a small size effect. Dark green and ** in the label indicate a moderate size effect. The third column shows responses when participants did the task with an autonomous virtual character in the *coupling* condition. The mean, 0.95 confidence intervals, and the extreme values are drawn as horizontal lines.

## Results

Participants were asked to perform circular movements with their hands for 1 min while trying to synchronise with a virtual character in front of them. First, they did a training trial where the virtual character facing them was animated by another participant. Then, three experimental trials followed where the virtual character was animated either by the movements of another participant (*human* condition), either with a Kuramoto oscillator^13^ with no coupling (*no coupling* condition) or with a small coupling factor (*coupling* condition). The order of these three experimental conditions was randomized. After each trial, participants were asked 10 questions exploring the self/other relation and the feeling of flow.

When comparing the *coupling* and *no coupling* conditions (see Table 1, Fig. 2) the questions that show significant differences (p < 0.05) and a moderate effect size reflect, for the *coupling* condition, a greater fluidity of the task, a greater influence of the participants’ own movements over the movements of the character in front, and a greater similarity with being in front of a mirror. Questions that show significant differences (p < 0.05) and a small effect size suggest that, for the *no coupling* condition, the character in front of them influenced their own movements more, that they felt more as if it was another person, and they felt less that the arms that moved were their own arms. We found no significant difference in concentration, challenge or time perception, nor in the influence of the other character on their movements. None of the questions showed a statistically significant difference between doing the task with an avatar driven by another human and with a completely autonomous virtual character with coupling (see Supplementary Table S1, Fig. 2). Responses to open questions in the post-experimental questionnaire (see Supplementary Data and Code) also suggest participants did not perceive differences between doing the task with another human or with an autonomous virtual character.

**Figure 3:**
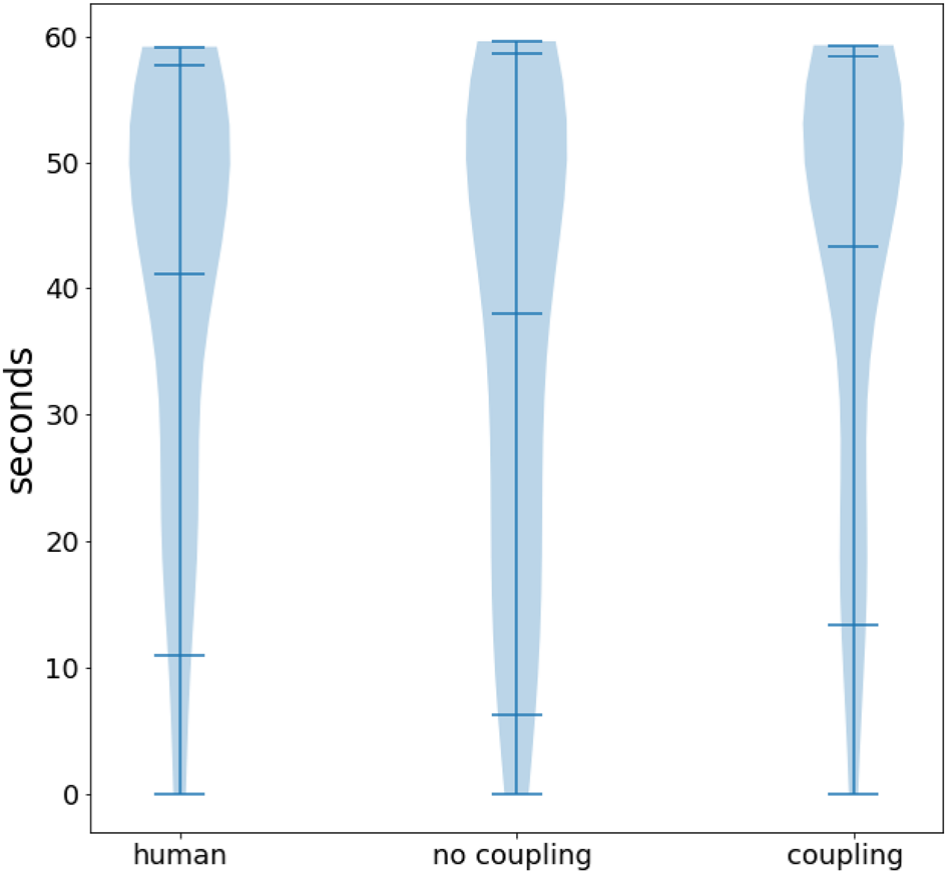
Amount of time participants reported the feeling of synchrony. The violin plots show the distribution in the amount of time that each participant reported feeling in synchrony for each condition. The mean, 0.95 confidence intervals, and the extreme values are drawn as horizontal lines.

Participants were also asked to report whether they felt in synchrony *during the trial*, through the buttons in their hand controllers. The amount of time in the one minute trial reporting the feeling, for each condition, is shown in Fig. 3. A Shapiro-Wilk test rejected the null hypothesis of normality for the different conditions (*human*: stats = 0.872 p = 0.001, *no coupling*: stats = 0.895 p = 0.003, *coupling*: stats = 0.822 p = 0.000). A Wilcoxon test comparing the sensation ratios in the *no coupling* and the *coupling* conditions did not find any significant difference (stats = 210, p = 0.135). Closer inspection of Fig. 3 shows a different distribution in the *no coupling* condition: the lower part of the distribution seems thicker than in the other two conditions.

It is possible that some users tend to press the buttons all the time, possibly due to the challenges associated with doing this task at the same time as mirroring the movements of the other. If this were the case, these participants would bias the entire statistical distribution towards appearing not normal. These responses would also hide any significant difference between the different conditions that did manage to report the feeling in real time. To take this possibility into account, we consider the condition *human* as a baseline. By doing this, the general tendency to press the button independently of the condition will be taken into account, and comparing *coupling-human* and *no coupling-human* will reflect the difference in perception between the two conditions. A Shapiro-Wilk test for both *coupling-human* and *no coupling-human* cannot reject the null hypothesis that these conditions are normally distributed (*coupling-human*: stats = 0.964 p = 0.319 and *no coupling-human*: stats = 0.939 p = 0.059). In the Supplementary Fig. S1 , the resulting distributions are shown. The data for individual subjects is shown in Supplementary Fig. S2. A Paired student test comparing the *coupling-human* condition (mean 2.217, std dev 16.464) with the *no coupling-human* condition (mean − 3.124, std dev 19.833 ) shows a significant difference ( Stats = 2.117 p = 0.042), which Clifford’s delta quantifies as a small effect.

## Discussion

In this work, we developed a full-body version of the mirror game^28,30^ and evaluated the subjective feeling of synchrony reported by people taking part in the mirror game, interacting with a virtual character that was either driven by another human or by a computational model. Results suggest that a relatively simple computational model of joint action, when used to drive the behaviour of an autonomous character in a joint action paradigm, induces a subjective sensation that is comparable to the experience of doing the task with a real human. Crucially, we did not find significant differences in any question between the *coupling* and *human* conditions.

Regarding differences between the *coupling* and *no coupling*, some items of the proposed survey that participants answered after each experimental condition highlight differences regarding fluency during the performance (Question 1), even if not regarding cognitive absorption in the activity (Questions 3 and 4). We believe the lack of significance in questions 3 and 4 may be related to the fact that the task was rather short.

Regarding the questions adapted from embodiment questionnaires, the coupling factor had a clear effect, as seen in questions 5 to 9. We did not notice a significant effect on question 10, which suggests no volitional motor contagion^31,32^, or a phenomenon that could be interpreted as a social version of the self-avatar follower effect^33^. The differences found in question 5 suggest that people felt differently about their virtual body in different conditions. However, it may also have been misinterpreted, and part of the participants might have understood that it referred to the arms of the character in front of them. Nevertheless, questions 6 and 9 consistently suggest that when the movements of the character in front of them were less coupled with their own, they felt as if the character was someone else. The reverse—that coupled movements tended to make people feel as if they were seeing themselves in front of a mirror—is consistent with the embodiment literature, where sensorimotor coupling is considered a crucial factor to induce the feeling of self-identification with a virtual body^34–36^. Before the experiment, we expected that for question 6 (“I felt as if the character in front of me was another person”) participants would implicitly assume the distinction *“it was a robot /it was another person”*. We were, therefore, expecting users would respond more towards *“it was another person”* in the cases where they did the task with the virtual character controlled by another person, as well as when they did the task in the coupling condition. However, users seem to implicitly have assumed the distinction “it was me/it was another person”. Consistently with this assumption, they answered more towards “it was another person” in the uncoupled condition, but not in the other two.

Responses to questions 7 and 8 suggest that in the *coupling* condition they perceived greater influence of their movements on the virtual character, as well as the need to make a greater effort to match the movement of the other in the *no coupling* one. The difference in these questions can also be interpreted from the perspective of who leads (question 7) and who follows (question 8), which are important in the mirror game literature^28,37–39^. In the *no coupling* condition participants were forced to follow the character, but in the *coupling* the situation was mixed: the coupling factor was small enough so that the virtual character would move even when the participant did not move. Therefore, in the *coupling* condition there was room for both following and leading with the movement. This seems to be reflected in the smaller effect size found in question 8, opposed to question 7, between the *coupling* and the *no coupling* conditions: participants reported more differences between conditions regarding how much their movements influenced the character, rather than regarding how much the movements of the character influenced their own movements.

The difference between conditions is also reflected in the amount of time people reported the feeling *at the same time* while they were doing the task (see Fig. 3). However, the results reflect a strong baseline bias. In the absence of repeated measures or control conditions like doing the task with another human, it may be more reliable to gather the subjective feeling of synchrony with post-trial questionnaires.

In the past, VR has been successfully used to study bodily self-consciousness, and has shown that sensorimotor correlations are crucial to feel a virtual body as our own^36,40–43^. From this perspective, the main difference between this scenario and previous experiments inducing self-identification with a virtual body is that here the coupling between the movements done and the movements seen (in the other character) is small. Indeed, if the coupling factors were strong, the character seen in front of each participant would be perceived as a virtual mirror, as often used to induce ownership over a virtual body^34,35^. The self-other distinction in this setup seems to appear because the coupling is small, or zero.

The responses to questions 6 to 9, adapted from embodiment questionnaires, suggest that virtual characters showing coupled behaviour with participants can be used to explore in a more nuanced way the distinction between ones’ bodily self and the body of others’, as well as to investigate the neural basis of interpersonal coordination and the motor planning and coordination related to joint action tasks. It can also be useful to study agency and virtual agency^44,45^.

Results also suggest a relation between the feeling of synchrony with an autonomous virtual character and the feeling of flow. The use of VR would bring better experimental control than with respect to current methods. It would also reduce the need to rely on the use of hyper-scanning techniques^46,47^. These require recording the brain activity of two or more people doing a joint task are recorded simultaneously, using either electroencephalography or functional Magnetic Resonance Imaging (fMRI), something that is far from being accessible to the wider neuroscience community.

Acknowledging the fact that the task was quite specific, this suggest relatively simple methods like the use of coupled oscillators are a viable strategy to create autonomous virtual characters that are perceived as more engaging by humans collaborating with them in VR. This strategy may open the door to the use of VR as a training tool for acquiring skills that require significant inter-subject coordination. Insofar, VR training for real world tasks has been demonstrated for activities that introduce significant physical constraints, such as billiards^48^ or table tennis^49^. Generalising the use of joint action computational models to a variety of tasks where inter-subject coordination plays a significant part of the task success could unlock the use of VR in a wider variety of training, education and therapy scenarios.

## Methods

### Participants

A total of 38 adult participants (among which 10 women) took part in the study. Two couples were excluded from the study. In one case, one of the participants never reported the feeling of synchrony. The other couple was excluded because of the malfunctioning of a hand controller. As a result, the data analysis involved 34 participants (10 women).

The experiment was approved by the Ethics committee of the University of Geneva, and all experiments were performed in accordance with the relevant regulations. All participants received an information sheet and an informed consent form, which they signed. They also received a financial compensation of 20 Swiss Francs for their participation.

### Experimental setup

The material used during the experiments included:

1. Two Head Mounted Displays (HMD), model Oculus Rift with handheld Oculus Touch Controllers.
2. Two PCs with n NVIDIA GeForce GTX 1080 graphics card, Intel Core i7 CPU (3.60 GHz), and 32 GB of RAM. Both machines ran on Windows 10.
3. The connection between the two computers was established through a wired Ethernet network.

The virtual environment was developed using Unity3D, version 2020.3.19f1. UDP was used for communication between the two machines and this communication was implemented with the standard .NET socket library.

### Experimental task

Upon arrival, participants were briefed and informed about the nature of the experiment in dyads, including the kind of movement they would have been asked to perform. After this, each participant was given an information sheet and an informed consent form to complete and sign. Once the informed consent form was signed, they were handed a Head Mounted Display (HMD) and two hand controllers. To isolate them acoustically from the environment, they were also equipped with headphones playing white noise.

The experiment was designed as a within-participant design. Inside the VR experience, a series of panels explained the task and gave instructions on where to stand. Participants were informed that they would be placed in front of a partner in the virtual environment and were asked to watch a fixation point, a red dot on the chest of the virtual character in front of them. An animation showed the movement to be performed. Specifically, they were asked to perform circular movements with their arms, palms facing outward. They were also told that both them and their partner should imitate each other. Then, the gender of the participant was asked. Based on their response, each participant was given a female or a male avatar. The height of each participant was automatically obtained from the position of the HMD and their avatar was scaled appropriately. Participants were then asked to look at their virtual palms to make sure they understood that the arms of the virtual body co-located with them matched their own movements. It was also a way to make sure participants were placed correctly with respect to their virtual avatar. In the last two panels, participants were introduced to the notion of synchrony and then they were instructed to push a button on the controller when they felt synchronisation during the interaction. Finally, participants were informed that the experiment consisted of a first training trial, followed by three other trials, all lasting 1 min.

When both participants finished reading the instructions and confirmed they were ready, the avatar of their partner appeared in front of them and the training trial started. After the training trial, they were reminded to look at the fixation point and to report when they felt in synchrony by holding down a button on the controller. Then, the following trial started in one of three conditions: *human*, *no coupling*, *coupling* (see “Stimuli” section, below, for further details on differences between conditions).

After each trial, participants were asked to answer a total of 10 randomised questions, on a Likert scale from − 3 to + 3. They were asked to answer within the VR, using their hand controller. Once the questionnaires were completed, they waited for a random amount of seconds sampled uniformly between 1 and 10 s or, if the *human* condition was selected, until the other participant had completed the previous task. Once they had completed the three trials and answered the corresponding questions, they removed the HMD and completed a short written questionnaire with open-ended questions.

### Questionnaires

Participants were asked to answer 10 graded questions after each trial (see Fig. 2 ), with a score between − 3 and + 3, presented in random order. The 10 graded questions were adapted from two existing questionnaires. First, the short flow scale, see the Appendix in ^50^, balancing questions focused on fluency of performance (questions 1, 2) and on cognitive absorption on activity (questions 3, 4). Second, we adapted embodiment questionnaires^51^ to explore embodiment and the self-other relation as typically perceived in an imitation task (questions 6 to 10). Participants were also asked to complete a written post-experimental questionnaire with the following open-ended questions:

1. In this experiment you did four trials. The first was a trial to check if you could do the task well. Then there were 3 more. Among these last 3 trials, did you notice any difference between the three trials?
2. What were the differences that you felt between the different trials?
3. Was there a particular trial that felt differently from the others two? If so, which one?
4. If there was one trial that felt differently, what was the difference?

The goal of the open-ended questions was to capture any additional subjective aspects that may have not emerged from the first questionnaire.

### Stimuli

The virtual characters shown in Fig. 1 were downloaded from the free repository Mixamo (https://www.mixamo.com/ ). The assets forming the virtual room were obtained from the Unity3D Asset store. For the character performing the task in front of the participants, both male and female versions were used to match the gender reported by the other participant. The characters were animated using an idle animation from Mixamo, over which the movements of the arms were animated using the Inverse Kinematics (IK) system built into Unity3D. The hand positions of the character interacting with the participant were modelled with two Kuramoto oscillators^13^, parameterized with features extracted from training trial data (see section Experimental task). The target positions for the right and left hands ($p_R(t)$ and $p_L(t)$) were calculated as a circular movement on the YZ plane, given by the following equations:

1a $$\begin{aligned}{}&p_R(t) = c_R + r_R(t) \cos(\omega _R(t) \times t ) \hat{z} + r_R(t) \sin(\omega _R(t) \times t) \hat{y} \end{aligned}$$

1b $$\begin{aligned}{}&p_L(t) = c_L - r_L(t) \cos(\omega _L(t) \times t) \hat{z} + r_L(t) \sin(\omega _L(t) \times t) \hat{y}, \end{aligned}$$

where $\hat{y}$ and $\hat{z}$ denote the unit vectors in directions y and z of the plane. *R* and *L* denote the right and left hands, respectively. $c_{R,L}$ is the average position of each hand of the other participant during the training trial. The radius for each hand at time *t* is updated with a noise term:

2a $$\begin{aligned}{}&r_{R}(t) = r_{R} (0) + n_{R}(t), \end{aligned}$$

2b $$\begin{aligned}{}&r_{L}(t) = r_{L} (0) + n_{L}(t), \end{aligned}$$

where $n_{R,L}(t)$ is a Perlin noise^52^ between $[- , 0.085, 0.085]$ with sampling frequency equal to 50*Hz*, and $r_{R,L}(0)$ is the average radius of the other participant during the training trial. Perlin noise was used instead of White noise to have a continuous noise signal (see also Table 2). The angular velocity is updated with the following equation:

3a $$\begin{aligned}{}&\omega _{L}(t) = \omega _{L}(t_{k-1}) + \dot{\omega }_{L}(t_k) \times \Delta t, \end{aligned}$$

3b $$\begin{aligned}{}&\omega _{R}(t) = \omega _{R}(t_{k-1}) + \dot{\omega }_{R}(t_k) \times \Delta t, \end{aligned}$$

where $\omega _{R,L}(0)$ is the average angular velocity of the other participant’s hand during the training trial (see also Table 2), ‘$t_k$’ and ‘$t_{k-1}$’ correspond to two successive simulation steps, and $\Delta t$ is the inverse of the sampling frequency) (50 Hz). The angular velocity dynamics is described by the formula:

4a $$\begin{aligned}{}&\dot{\omega }_{R}(t) = K_{inter} \times \sin (\theta _{L}^{H}(t) - \theta _{R}^{V}(t)) + K_{intra} \times \sin (\theta _{L}^{V}(t) - \theta _{R}^{V}(t)), \end{aligned}$$

4b $$\begin{aligned}{}&\dot{\omega }_{L}(t) = K_{inter} \times \sin (\theta _{R}^{H}(t) - \theta _{L}^{V}(t)) + K_{intra} \times \sin (\theta _{R}^{V}(t) - \theta _{L}^{V}(t)), \end{aligned}$$

where *H* and *V* denote the human and the virtual character, respectively. $\theta (t)$ is the phase at time *t* of the corresponding hand. $K_{intra}$ is the intra-subject coupling factor, fixed at 0.005. $K_{inter}$ is the inter-subject coupling factor, and it will depend on the condition (see below).

**Table 2:** The values of the model parameters estimated in the training condition.

Parameter	Value (mean $\pm$ std)
$r_{R}(0)$	$0.163 \pm 0.049 \,[\text{m}]$
$r_{L}(0)$	$0.161 \pm 0.046\, [\text{m}]$
$\omega _R(0)$	$2.695 \pm 1.025 \,[\text{s}^{-1}]$
$\omega _L(0)$	$2.698 \pm 1.025\, [\text{s}^{-1}]$

The previous computational model was used differently in the three experimental conditions:

1. in the *human* condition, the hand position of the other participant was used as target position, and the computational model was not used;
2. in the *no coupling* condition, the model was used with a null $K_{inter}$. The position of the YZ plane were the hands moved was fixed at 0.343 m from the position of the character;
3. in the *coupling* condition, the model was used with a $K_{inter}$ equal to 0.0075. The value was adjusted manually where the influence of the participant’s movement could be felt but it did not feel like a virtual mirror. In addition, with this value the autonomous character would still move when the player did not, helping to not give away the fact that it was an autonomous virtual character, and not a human participant. The position of the YZ plane was also fixed at 0.343.

In each of the three conditions, the virtual character in front of them was placed at 1.246 m from the participant. To help differentiate each trial, the virtual character in front of them had a sweater of a different colour (white, pink, green or blue) each time.

### Data analysis

Questionnaire responses were analysed using non-parametric Wilcoxon tests^53^. The reports of subjective synchrony were summarised as the amount of time participants reported the feeling in each condition. Effect sizes were estimated using Clifford’s delta^54^. The resulting metrics were tested for normality using the Shapiro–Wilk test^55^. When normality could not be assumed we compared conditions with Wilcoxon tests. When it could be assumed we compared conditions with paired-samples Student t-tests. All the statistical tests used were imported from the Python scipy toolkit, to the exception of Cifford’s delta, which used the implementation available in https://github.com/neilernst/cliffsDelta. All the data and Python scripts used for the analysis are available in the Supplementary Data and Code.

## Supplementary Information

Supplementary Video 1.

Supplementary Information 1.

Supplementary Information 2.

## Data Availability

All data generated during this study and the scripts to analyse them are included in this published article and its Supplementary Information files.
