# Supplementary material for: Playing the mirror game in virtual reality with an autonomous character
Source: Sci Rep. 2022 Dec 9;12:21329. doi: 10.1038/s41598-022-25197-z (PMC9734752; doi:10.1038/s41598-022-25197-z)
Supplement: Supplementary file 3 — Supplementary Information 2. [file 41598_2022_25197_MOESM3_ESM.pdf]

Playing the Mirror Game in Virtual Reality with an Autonomous Character

Joan Llobera[1,*], Valentin Jacquat[1], Carmela Calabrese[2], and Caecilia Charbonnier[1,3]

[1]Artanim Foundation, Meyrin, 1217, Switzerland.
[2]Department of Electrical Engineering and Information Technology, University of Naples Federico II, 80125 Naples, Italy.
[3]University of Geneva, Faculty of Medicine, Geneva, 1211, Switzerland.
[*]joan.llobera@artanim.ch

ABSTRACT

Perceptual-motor synchronisation in human groups is crucial in many activities, from musical ensembles to sports teams. To this aim, the mirror game, where partners are asked to imitate each other's movements or gestures, is one of the best available experimental paradigms to study how humans engage in joint tasks and how they tend to synchronise their behaviour. However, to date, virtual reality characters do not engage in motor synchronisation with human users. In this work, we explored to what extent an autonomous virtual character and a human that play the mirror game in a virtual reality can synchronise their behaviour. We created a full-body version of the mirror game with an autonomous virtual character, whose movements were driven by a model based on coupled oscillators. Participants engaged in a joint imitation task with a virtual player animated with three options: a model that included a small coupling, a model with no coupling, or another human. Behavioural measures and subjective reports suggest that participants were unable to distinguish the condition of small coupling from the engagement with an avatar driven by another human participant.

Supplementary tables and figures

Question: During the last trial...	**coupling**	**human**	Z score	p	*d*	effect size
1. I did the task fluidly and smoothly	1.2	1.4	93.5	0.665	0.1	
2. I had no problem to concentrate during the task	1.4	1.2	93.5	0.662	-0.1	
3. I felt just the right amount of challenge	1.0	1.2	90.5	0.854	0.1	
4. I did not notice time passing	1.2	1.0	85.5	0.462	-0.0	
5. I felt like the arms that moved when I moved were my own arms	1.8	1.9	40.0	0.698	-0.0	
6. I felt as if the character in front of me was another person	0.4	0.7	77.0	0.466	0.1	
7. my movements influenced the movements of the character that was in front of me	1.3	0.6	146.0	0.192	-0.3	
8. the movements of the character in front of me influenced my own movements	0.9	0.9	102.5	0.925	-0.0	
9. the character in front of me moved exactly like me, as if I was looking at a mirror	0.3	0.1	147.0	0.675	-0.1	
10. when the character in front of me moved, I felt the instinct to move	1.0	1.1	119.0	0.561	0.1	

Table S1. Wilcoxon tests comparing the graded responses to the **coupling** condition with the graded responses to the **human** condition. The values reported are the median values for each condition, the Z score, the p value and Cliff's delta. Since no test gives a significant effect ($p<0.05$), the effect size is not reported.

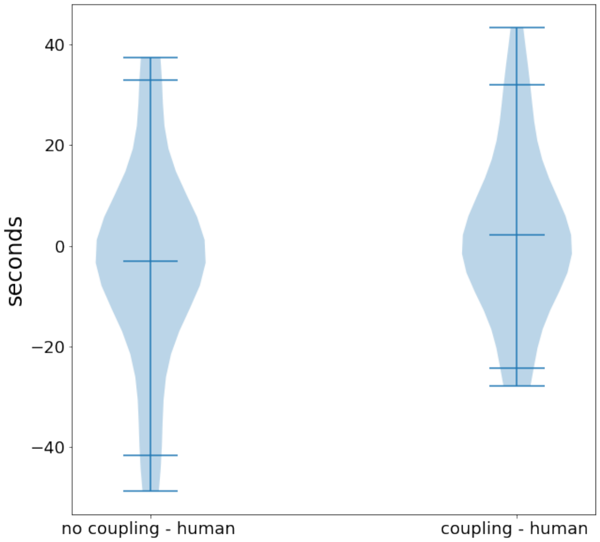

Figure S1. Violin plots of the conditions **coupling** and **no coupling** when considering the **human** condition as a baseline. The mean, .95 confidence intervals, and the extreme values are drawn as horizontal lines.

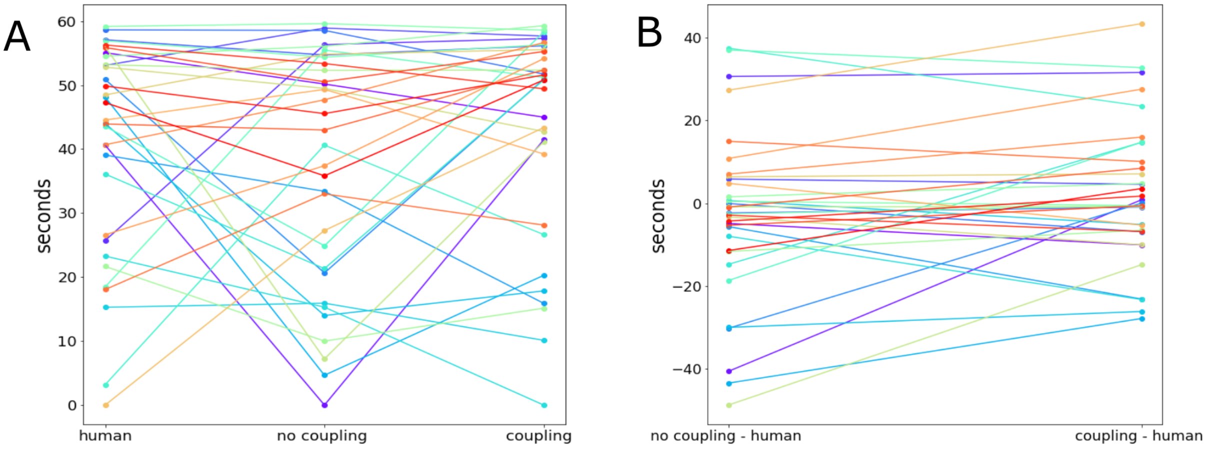

Figure S2. Individual plots of the amount of time that participants pressed the button reporting synchrony. In A we show the amount of time they pressed the button separately per condition. In B we show the amount of time they pressed the button considering the **human** condition as a baseline
